# Clinician Burnout Associated With Sex, Clinician Type, Work Culture, and Use of Electronic Health Records

**DOI:** 10.1001/jamanetworkopen.2021.5686

**Published:** 2021-04-20

**Authors:** Eugenia McPeek-Hinz, Mina Boazak, J. Bryan Sexton, Kathryn C. Adair, Vivian West, Benjamin A. Goldstein, Robert S. Alphin, Sherif Idris, W. Ed Hammond, Shelley E. Hwang, Jonathan Bae

**Affiliations:** 1 Duke University Health System, Durham, North Carolina; 2 Department of Psychiatry and Behavioral Sciences, Duke University School of Medicine, Durham, North Carolina; 3 Department of Biostatistics and Bioinformatics, Duke University School of Medicine, Durham, North Carolina; 4 Duke Center for Healthcare Safety and Quality, Duke University Health System, Durham, North Carolina; 5 Duke Center for Health Informatics, Duke Clinical and Translational Science Institute; Duke University, Durham, North Carolina; 6 Duke University School of Medicine, Durham, North Carolina; 7 Department of Community and Family Medicine, Duke University School of Medicine, Durham, North Carolina; 8 Department of Surgery, Duke University School of Medicine, Durham, North Carolina; 9 Department of Medicine, Duke University School of Medicine, Durham, North Carolina

## Abstract

**Importance:**

Electronic health records (EHRs) are considered a potentially significant contributor to clinician burnout.

**Objective:**

To describe the association of EHR usage, sex, and work culture with burnout for 3 types of clinicians at an academic medical institution.

**Design, Setting, and Participants:**

This cross-sectional study of 1310 clinicians at a large tertiary care academic medical center analyzed EHR usage metrics for the month of April 2019 with results from a well-being survey from May 2019. Participants included attending physicians, advanced practice providers (APPs), and house staff from various specialties. Data were analyzed between March 2020 and February 2021.

**Exposures:**

Clinician demographic characteristics, EHR metadata, and an institution-wide survey.

**Main Outcomes and Measures:**

Study metrics included clinician demographic data, burnout score, well-being measures, and EHR usage metadata.

**Results:**

Of the 1310 clinicians analyzed, 542 (41.4%) were men (mean [SD] age, 47.3 [11.6] years; 448 [82.7%] White clinicians, 52 [9.6%] Asian clinicians, and 21 [3.9%] Black clinicians) and 768 (58.6%) were women (mean [SD] age, 42.6 [10.3] years; 573 [74.6%] White clinicians, 105 [13.7%] Asian clinicians, and 50 [6.5%] Black clinicians). Women reported more burnout (survey score ≥50: women, 423 [52.0%] vs men, 258 [47.6%]; *P* = .008) overall. No significant differences in EHR usage were found by sex for multiple metrics of time in the EHR, metrics of volume of clinical encounters, or differences in products of clinical care. Multivariate analysis of burnout revealed that work culture domains were significantly associated with self-reported results for commitment (odds ratio [OR], 0.542; 95% CI, 0.427-0.688; *P* < .001) and work-life balance (OR, 0.643; 95% CI, 0.559-0.739; *P* < .001). Clinician sex significantly contributed to burnout, with women having a greater likelihood of burnout compared with men (OR, 1.33; 95% CI, 1.01-1.75; *P* = .04). An increased number of days spent using the EHR system was associated with less likelihood of burnout (OR, 0.966; 95% CI, 0.937-0.996; *P* = .03). Overall, EHR metrics accounted for 1.3% of model variance (*P* = .001) compared with work culture accounting for 17.6% of variance (*P* < .001).

**Conclusions and Relevance:**

In this cross-sectional study, sex-based differences in EHR usage and burnout were found in clinicians. These results also suggest that local work culture factors may contribute more to burnout than metrics of EHR usage.

## Key Points

**Question:**

What is the association of clinician sex, use of the electronic health record (EHR), and work culture with clinician burnout?

**Findings:**

This cross-sectional study of 1310 clinicians found burnout to be more prevalent in women, attending physicians, and advanced practice providers. Multivariate modeling of burnout identified local work culture accounting for 17.6% variance compared with only 1.3% variance for EHR metrics. Female sex independently contributed more to likelihood of clinician burnout and significantly interacted with work culture domains of commitment and work-life balance.

**Meaning:**

These findings suggest that clinician sex and local work culture may contribute more to burnout than the EHR.

## Teaser

This cross-sectional study of clinicians at a large tertiary care academic medical center examines the association of clinician burnout with sex, clinician type, work culture, and use of electronic medical records.

## Introduction

Recognition of burnout among health care clinicians has increased over the past 10 years, the same timeframe over which electronic health records (EHRs) have been rapidly adopted.^1,2,3,4^ The negative effects of burnout extend beyond the well-being of clinicians themselves to include clear correlations with increased errors and poorer outcomes for their patients.^5,6,7^ Health care worker burnout has become a significant focus of research with specific attention to the EHR as a contributing factor.^8,9,10,11,12^ Differentiating the potential contribution of the EHR to clinician burnout provides opportunities for better interventions.

Changes in care processes introduced with the EHR include increased time spent completing clinical work, especially after scheduled work hours.^13,14,15^ In their 2017 study, Arndt et al^16^ demonstrated with time-and-motion studies of clinical care and EHR usage metrics that clinicians spend 5.9 hours in the EHR out of an 11.4-hour day. Another study found that physicians spend an average 1 to 2 hours in the EHR after hours per scheduled day.^17^

Variations by sex in clinical care and usage of the EHR are also becoming more apparent. A 2020 study^18^ demonstrated that female primary care clinicians spent more time with their patients at the point of clinical care, and a 2017 study^19^ found that female hospitalists’ patients experience lower mortality and fewer readmissions. Differences in EHR usage by sex have identified that female clinicians spend more time in the EHR overall.^20,21^ It has also been shown that female clinicians have more burnout than their male counterparts.^22,23^ These differences across clinical care, patient outcomes, burnout, and EHR usage are described primarily for attending physicians. There is limited literature to evaluate the sex differences for alternate clinician groups.

Much of the literature on clinician burnout is in the form of surveys of burnout and perceived burdens of the EHR.^24,25^ EHR usage logs provide quantifiable data demonstrating clinician time and volume of activities in the EHR and provides an opportunity to differentiate usage patterns between user groups. Time-and-motion studies of clinical care and EHR usage metadata have validated the correlation of these metrics as a good proxy of clinician activities.^26^ Nevertheless, a recent systematic analysis of EHR metrics found a need for these studies to better define EHR metrics in standard methodologically transparent formats.^27^ The purpose of this study was to describe clinician burnout using clinician demographic characteristics, EHR usage, and surveys of local work culture.

## Methods

This cross-sectional study included 3 types of clinicians using EHR usage metadata metrics and an institutional survey of burnout, wellness, and work satisfaction. This study was reviewed by the Duke University institutional review board and deemed exempt from informed consent requirements because data were deidentified.

### Participants and Data Source

**Figure 1.**
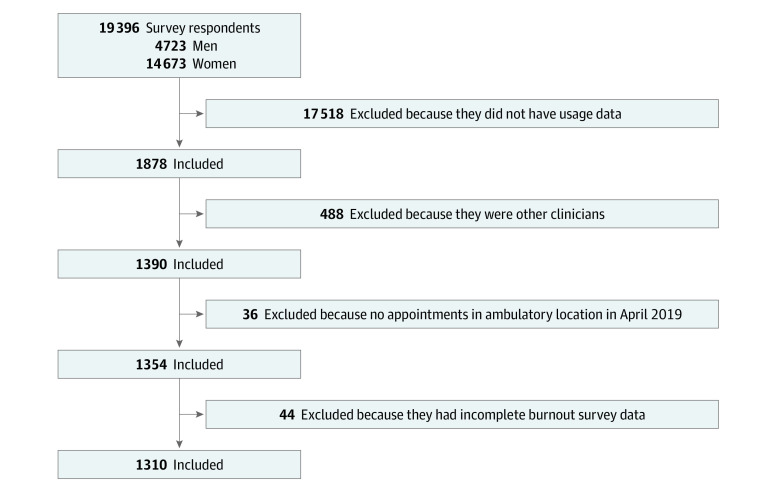
Cohort Development Flow Diagram

Clinicians included in the study practice in primarily outpatient settings in an academic tertiary health care system. We collected data for clinicians who had participated in an institution-wide employee engagement and work culture survey in mid-May 2019 (19 396 individuals with a response rate of 72.3%). Participants were restricted to 3 types of clinicians: attending physicians, advanced practice providers (APPs), and house staff with at least 1 day of outpatient appointments for the month of April 2019 and who had complete burnout survey responses. Of these, 1848 individuals had EHR usage metadata for the study period of April 2019 and 1310 met our inclusion/exclusion criteria (Figure 1).

We used EHR usage metadata from our vendor’s EHR usage report (called Signal [Epic]). EHR usage metrics are derived from a user’s interactions with the EHR that are captured in the User Action Log (UAL) Lite. The UAL calculates active time in EHR activity based on keyboard clicks or any mouse movements. After 5 seconds of inactivity, attribution of active time capture ceases. Time in the system during scheduled hours is measured as 30 minutes before the first patient and 30 minutes after the last patient. The Signal report divides time in the EHR to time spent within scheduled hours, time outside of scheduled hours, and time on nonscheduled days without appointments.

We chose EHR usage data for the study period of April 2019 for its temporal correlation with the May 2019 wellness/burnout survey. We used metrics for total time in the EHR and volume of EHR usage for clinical activities of patient encounters, in-basket messages, and documentation. We evaluated 9 EHR metrics directly and derived an additional 7 metrics to enable evaluations across all clinicians (eTable 1 in the Supplement includes operational definitions and EHR metric). The calculated EHR metrics include total ambulatory encounters (15 metrics), total in-basket messages received (109 metrics), and proportional metric of time spent in the EHR (after hours/total hours). For the purposes of consolidating discussion of nonscheduled time, *after hours* henceforth refers to a summation measure of time spent working in EHR after work hours on scheduled days plus time spent working on nonscheduled days.

### Well-being Survey Data

An organizational employee work culture survey was administered to the entire health system in mid-May 2019 and received responses from 19 396 individuals (72.3%). This survey is administered periodically for our health system by a third-party vendor to maintain respondent anonymity.^28^ EHR usage metadata was linked to the institutional survey using unique user identification, respondent identifiers were removed, and data returned for further analysis. The demographic variable for sex (male or female) was self-identified at the beginning of employment.

The survey includes a 5-item derivative of the Maslach Burnout Inventory emotional exhaustion domain (henceforth *burnout*).^29,30,31,32,33,34^ While the Maslach Burnout Inventory is the gold standard for burnout measurement, a meta-analysis found that the other 2 domains of burnout, depersonalization and personal accomplishment, consistently produced smaller coefficient α estimates than emotional exhaustion.^35^ In addition, emotional exhaustion is more psychometrically robust in discriminating between burnout and nonburnout outpatients suffering from work-related neurasthenia.^36^^.^

The survey also includes additional work culture domains such as commitment, belonging, safety, teamwork, and work-life balance. The surveys are set to a 5-point Likert response (from 1 = “strongly disagree” to 5 = “strongly agree”). Where appropriate, survey results are reverse scored to account for item valence, such that low domain scores always represent negative outcomes and high domain scores represent positive outcomes. We averaged Likert results from all questions in a subdomain to determine a representative score for that response. Measures, their definitions, and Cronbach’s α values are reported in eTable 2 in the Supplement.

Burnout is transformed to a 100-point scale (0-100) for ease of interpretation, with higher scores representing more burnout. For purposes of graphical visualizations, we categorized burnout into 4 groups—none (0-49), mild (50-74), moderate (75-99), and severe (100) burnout—representing a burnout spectrum.^37,38^

### Statistical Analysis

We used standard descriptive statistics to summarize clinician demographic characteristics, a representative subset of EHR usage metrics, and wellness survey responses. We found all data to be nonparametric and report out summary values as medians with interquartile ranges (IQRs). Work culture survey responses are presented as mean values with standard deviations (SDs) to preserve meaningful variations, which are less evident with IQRs reported as Likert scale results.

We conducted a multivariate analysis to assess the simultaneous association of burnout with clinician demographic characteristics, EHR usage metrics, and well-being domains. For this analysis, burnout was dichotomized using a score of 50 or greater as evidence of burnout.^13^ We used logistic regression to assess the likelihood of burnout occurring given the covariates in the model.^39^ The parameter coefficient estimates were converted to odds ratios (OR) for ease of interpretation. Model fit statistics were assessed using McFadden pseudo *R^2^* and the likelihood ratio χ^2^ test of the fitted vs the intercept model.^40^ We conducted a hierarchal assessment using different permutations of covariates analyzed separately by clinician demographic characteristics, EHR metrics, and wellness survey domains to determine variables with the strongest contribution to measures of burnout. Race/ethnicity was examined for inclusion in the regression model but was not included because there was no variation of burnout in other racial/ethnic categories other than White. We completed an interaction analysis of sex with other model covariates to help define the relationship sex has on burnout given levels of other metrics. The Akaike information criteria was used as a relative fit statistic for model comparison.^41^ Finally, we completed likelihood ratio tests to examine the significance of variance explained by the contributions between demographic characteristics, EHR metrics, and wellness domain blocks to the final model.

Statistical analysis was conducted with STATA/SE version 16.1 (StataCorp LLC). Significance was set at α < .05.

## Results

Of the 1310 clinicians included for analysis, 542 (41.4%) were men (mean [SD] age, 47.3 [11.6] years; 448 [82.7%] White clinicians, 52 [9.6%] Asian clinicians, and 21 [3.9%] Black clinicians) and 768 (58.6%) were women (mean [SD] age, 42.6 [10.3] years; 573 [74.6%] White clinicians, 105 [13.7%] Asian clinicians, and 50 [6.5%] Black clinicians). Further information on demographic characteristics, specialty, and wellness survey responses are presented in Table 1.

**Table 1.** Clinician Demographic Characteristics and Survey Responses by Sex

Characteristic	No. (%)	
	Male	Female
Clinician type	542 (41.4)	768 (58.6)
Attending	353 (65.1)	499 (65.0)
APPs	144 (26.6)	216 (28.1)
House staff	45 (8.3)	53 (6.9)
Age, mean (SD), y	47.3 (11.6)	42.6 (10.3)
Burnout (survey score ≥50)	258 (47.6)	423 (55.1)
Race/ethnicity^a^		
White, non-Hispanic	448 (82.7)	573 (74.6)
Asian	52 (9.6)	105 (13.7)
Black/African American	21 (3.9)	50 (6.5)
Other^b^	21 (3.9)	40 (5.2)
Practice type		
Surgery/anesthesia	157 (29.0)	254 (33.1)
Medicine	134 (24.7)	184 (24.0)
Primary care	115 (21.2)	162 (21.1)
Psychology/Neurology	64 (11.8)	94 (12.2)
Pediatrics	58 (10.7)	55 (7.2)
Radiology/radiation oncology	14 (2.6)	19 (2.5)
Survey responses, mean (SD)^c^		
Burnout	45.8 (23.1)	51.0 (22.3)
Belonging	3.92 (.883)	3.81 (.978)
Diversity	4.03 (1.01)	3.89 (1.00)
Well-being support	4.07 (.787)	4.04 (.809)
Career development	3.73 (.872)	3.69 (.806)
Commitment	3.89 (.801)	3.81 (.876)
Empowerment	3.90 (.782)	3.85 (.837)
Management	3.83 (.825)	3.80 (.885)
Safety	4.06 (.654)	3.99 (.705)
Teamwork	4.25 (.707)	3.95 (.874)
Violence	3.64 (.938)	3.59 (1.00)
Work life	3.73 (.988)	3.61 (1.10)

Abbreviation: APPs, advanced practice providers.

^a^ Demographic data derived from initial employment self-identification.

^b^ Racial/ethnic groups classified as other included American Indian or Alaskan native, Hispanic, Native American or other Pacific Islander, and identifying as 2 or more.

^c^ Wellness Survey definitions and Cronbach α reported in eTable 2 in the Supplement.

Female clinicians reported more burnout than their male counterparts (score ≥50, median [IQR] percentage: men, 45% [30%-60%] vs women, 50% [35%-70%]; *P* < .001) (Table 2). Analysis of burnout by sex and clinician type found significant differences for attending physicians (men, 45% [30%-65%] vs women, 50% [35%-70%]; *P* < .001) and APPs (men, 35% [25%-60%] vs women, 45% [35%-60%]; *P* = .03) but not house staff (men, 55% [35%-75%] vs women, 50% [35%-75%]; *P* = .89).

**Table 2.** EHR Usage Metrics for April 2019 by Sex^a^

Wellness survey metric	EHR metric score, median (IQR)		*P* value
	Male	Female	
Burnout survey score value (continuous variable)	45 (30 to 60)	50 (35 to 70)	<.001^b^
Attending	45 (30 to 65)	50 (35 to 70)	<.001
APPs	35 (25 to 60)	45 (30 to 60)	.03
House staff	55 (35 to 75)	50 (35 to 75)	.89
**Signal metrics for time and patients**			
Patient age, y^c^	53.2 (35.7 to 60.4)	52.8 (39.7 to 60.3)	.43^b^
Attending	53.9 (34.7 to 60.8)	53.7 (40.4 to 60.8)	
APPs	53.2 (40.1 to 60.5)	54.2 (41.8 to 60.5)	
House staff	42.3 (29.0 to 57.1)	42.3 (30.9 to 53.2)	
Total time in EHR, min^c^	1551 (748 to 2750)	1780 (792 to 3041)	.14^b^
Attending	1326 (602 to 2546)	1476 (602 to 2630)	
APPs	2494 (1650 to 3359)	2746 (1887 to 3529)	
House staff	810 (507 to 1269)	927 (666 to 1394)	
Total days in EHR, d^c^	18 (13 to 22)	18 (14 to 21)	.41^b^
Attending	19 (14 to 22)	19 (14 to 22)	
APPs	17.5 (12 to 20.5)	18 (15 to 20)	
House staff	12 (9 to 19)	15 (9 to 20)	
Calculated total unscheduled time, min^c^	435 (89 to 876)	480 (123 to 1015)	.19^b^
Attending	457 (170 to 869)	464 (163 to 942)	
APPs	480 (68 to 999	591 (138 to 1277)	
House staff	0 (0 to 438)	0 (0 to 597)	
Proportion of after-hours time by total time in EHR^c^	30.6 (5.8 to 49.9)	30.5 (8.9 to 52.3)	.63^b^
Attending	36.1 (15.0 to 52.4)	33.5 (14.1 to 54.1)	
APPs	18.4 (3.4 to 39.4)	21.8 (6.7 to 42.3)	
House staff	0 (0 to 42.1)	0 (0 to 55.6)	
**Clinical volume metrics**			
Total days with appointments^c^	9 (5 to 14)	11 (5 to 15)	.09^b^
Attending	9 (5 to 14)	10 (5 to 15)	
APPs	12 (8 to 15)	12 (9 to 15)	
House staff	3 (2 to 4)	3 (2 to 5)	
Total encounters for mo^c^	43 (9 to 104)	48 (12 to 112)	.32^b^
Attending	45 (13 to 103)	47 (13 to 103)	
APPs	66.5 (13.5 to 138)	71 (23.5 to 143)	
House staff	5 (2 to 11)	7 (−3 to 13)	
Progress note length, No. of characters^c^	6586 (4215 to 8836)	6482 (4589 to 9453)	.37^b^
Attending	6166 (3885 to 8427)	6242 (4087 to 9168)	
APPs	7318 (5387 to 9748)	6958 (5050 to 10 550)	
House staff	7283 (5759 to 9418)	7135 (5901 to 9136)	
Charts closed same day, %^c^	70.0 (33.3 to 95.0)	69.7 (35.5 to 93.5)	.92^b^
Attending	69.8 (33.3 to 94.5)	67.6 (36.0 to 93.3)	
APPs	84.6 (54.5 to 98.8)	83.1 (54.5 to 97.0)	
House staff	11.0 (0 to 43.0)	6.7 (0 to 28.0)	
**In-basket metrics**			
Total in-basket messages received, No./mo^c^	298.5 (115 to 534)	273 (112 to 498.5)	.82^b^
Attending	348 (133 to 579)	292 (133 to 564)	
APPs	285.5 (124 to 523.5)	306 (158 to 463)	
House staff	75 (39 to 167)	70 (43 to 126)	
Time per completed message, s^c^	39.6 (25.1 to 61.8)	39.8 (23.9 to 63.7)	.75^b^
Attending	34.3 (21.5 to 50.5)	34.3 (22.3 to 56.2)	
APPs	53.3 (37.0 to 83.4)	53.6 (34.4 to 84.0)	
House staff	43.7 (20.7 to 76.9)	44.4 (23.0 to 66.1)	

Abbreviations: APPs, advanced practice providers; EHR, electronic health record; IQR, interquartile ranges.

^a^ eTable 1 in the Supplement includes definitions for EHR metrics (direct and calculated).

^b^ Mann-Whitney U inter-sex testing.

^c^ EHR metrics both direct and derived.

We found nonsignificant differences with EHR usage by sex for related clinical time and volume activities. Female clinicians spent more time in the EHR by total time in minutes (median [IQR] minutes: men, 1551 [748-2750] vs women, 1780 [792-3041]; *P* = .14) but not more days in the EHR (median [IQR] days: men, 18 [13-22] vs women, 18 [14-21]; *P* = .41). Metrics for volume of clinical work showed that female clinicians had more days with appointments (median [IQR] days: men, 9 [5-14] vs women, 11 [5-15]; *P* = .09) and more clinical encounters (median [IQR] total encounters: men, 43 [9-104] vs women, 48 [12-112]; *P* = .32), although these differences were not statistically significant. Female clinicians received less in-basket messages compared with male clinicians (median [IQR] messages/mo: men, 298.5 [115-534] vs women, 273 [112-498.5]; *P* = .82) but the difference was not statistically significant. There were no differences in products of clinical encounters, including length of documentation or percentage of encounters closed the same day.

**Figure 2.**
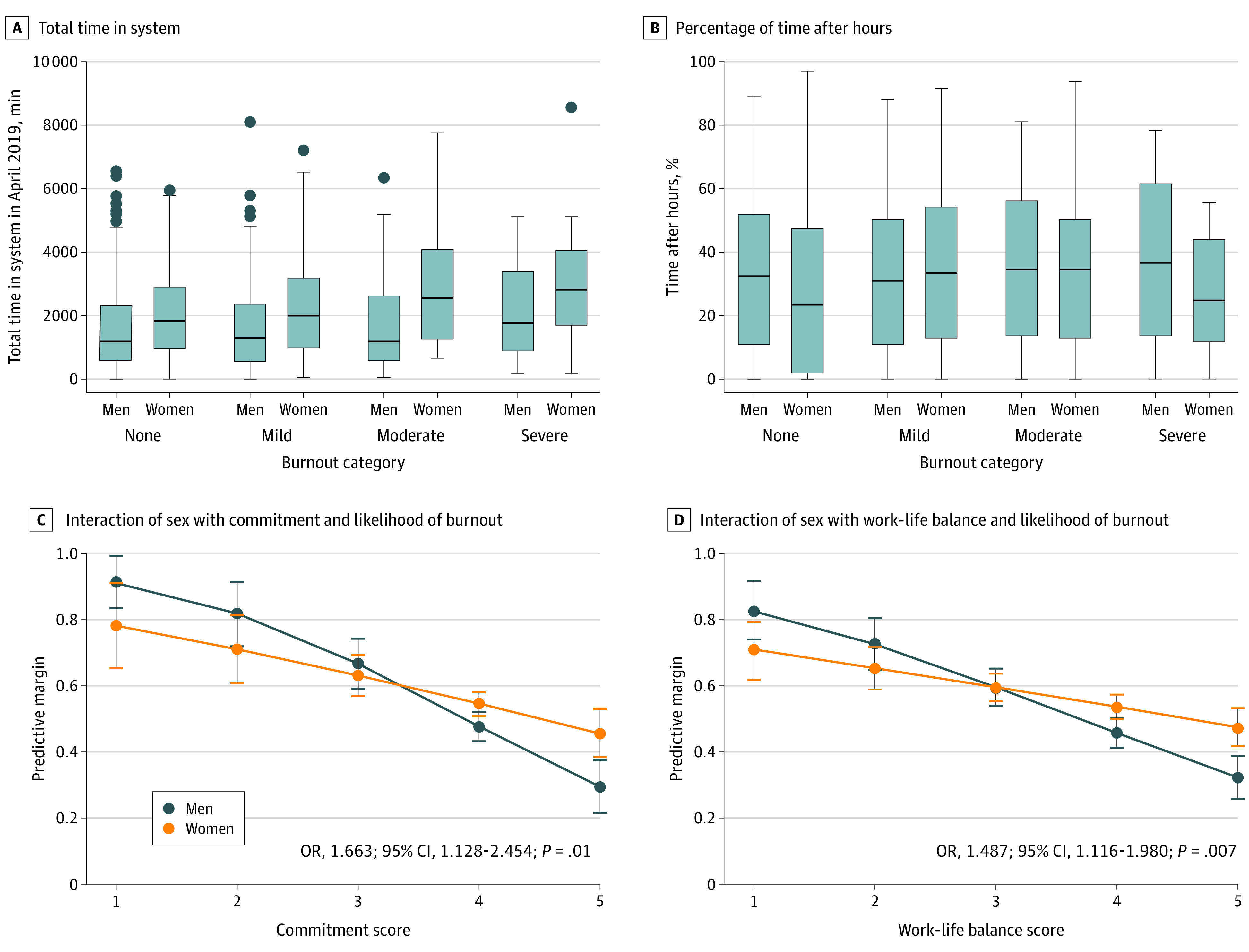
Graphical Data Visualizations of Time EHR Metrics and Interaction of Sex in Full Model

To evaluate whether increased total time in the EHR correlated with increased after-hours time, we examined the percentage of time spent after hours by sex and burnout category. We found no difference in the percentage of time spent after hours (median [IQR] percentage: men, 30.6% [5.8%-49.9%] vs women, 30.5% [8.9%-52.3%]; *P* = .63). Regardless of level of burnout or sex, all clinicians spent similar time in the EHR after hours (Figure 2). Surprisingly, female clinicians with moderate to severe burnout spent a smaller proportion of time after hours than equivalently burned-out males.

### Burnout Logistic Regression Results

We conducted a logistic regression model to assess the association between well-being domains and EHR usage metrics with clinician burnout. The parameter estimates are presented in Table 3. The model fit statistics showed an adequate fit of the data. The likelihood ratio χ^2^ test was significant (χ^2^_20_ = 319.82; *P* < .001; McFadden *R^2^* = 0.198).

**Table 3.** Multivariate Logistic Regression Models of Clinician Demographics, EHR Metrics, and Well-being Survey Domains to Burnout

Characteristics	Model 1 (clinician demographics), OR (95% CI)	*P* value	Model 2 (model 1 + EHR metrics), OR (95% CI)	*P* value	Model 3 (model 2 + well-being metrics), adjusted OR (95% CI)	*P* value
Clinician sex	1.404 (1.112-1.762)	.003	1.424 (1.132-1.792)	.003	1.331 (1.010-1.754)	.04
Clinician age	1.005 (0.995-1.016)	.29	1.005 (0.995-1.016)	.31	1.008 (0.996-1.020)	.20
Average patient age	0.992 (0.986-0.998)	.01	0.989 (0.983-0.995)	<.001	0.993 (0.985-1.001)	.07
Specialty	1.056 (0.987-1.131)	.11	1.046 (0.972-1.124)	.23	1.054 (0.964-1.151)	.25
Days in EHR for month	NA	NA	0.979 (0.955-1.003)	.09	0.966 (0.937-0.996)	.03
Total time in system	NA	NA	1.000 (1.000-1.003)	.002	1.000 (1.000-1.000)	.07
Days with appointment	NA	NA	0.987 (0.955-1.019)	.43	1.003 (0.963-1.046)	.88
Total encounters	NA	NA	0.998 (0.996-1.000)	.11	1.000 (0.998-1.003)	.76
Total in-basket messages	NA	NA	1.001 (1.000-1.001)	.02	1.000 (1.000-1.000)	.19
Commitment	NA	NA	NA	NA	0.542 (0.427-0.688)	<.001
Work life	NA	NA	NA	NA	0.643 (0.559-0.739)	<.001
Belonging	NA	NA	NA	NA	0.822 (0.665-1.017)	.07
Teamwork	NA	NA	NA	NA	0.525 (0.409-0.672)	<.001
Empower	NA	NA	NA	NA	0.929 (0.729-1.184)	.55
Management	NA	NA	NA	NA	1.008 (0.811-1.251)	.95
Career development	NA	NA	NA	NA	1.017 (0.827-1.250)	.87
Safety	NA	NA	NA	NA	1.129 (0.853-1.494)	.40
Diversity	NA	NA	NA	NA	0.837 (0.710-0.985)	.03
Well-being	NA	NA	NA	NA	0.883 (0.740-1.053)	.17
Violence	NA	NA	NA	NA	1.192 (0.985-1.441)	.07
No.	1310	NA	1310	NA	1167	NA
χ^2^	χ^2^ _4_ = 18.33	.001	χ^2^ _9_ = 41.02	<.001	χ^2^ _20_ = 319.82	<.001
McFadden *R^2^*	.010	NA	.023	NA	.198	NA
AIC	1.38	NA	1.37	NA	1.147	NA
Δ Variance M1 to M2	1.3%	NA	NA	.001	NA	NA
Δ Variance M2 to M3	NA	NA	17.6%	NA	NA	<.001

Abbreviations: AIC, Akaike information criteria; EHR, electronic health record; NA, not applicable; OR, odds ratio.

The results of the model indicate sex, total days in the EHR, and 4 survey domains were predictive of burnout. Holding all other variables in the model constant, female clinicians had an increased likelihood of burnout overall (OR = 1.331; 95% CI, 1.010-1.754; *P* = .04). As total number of days in the EHR increased, the likelihood of burnout modestly decreased (OR = 0.966; 95% CI, 0.937-0.996; *P* = .03). We found no other EHR metrics to be statistically significant in the full model. However, several of the wellness survey domains were significant. The results show that as the level of commitment increased, the likelihood of burnout decreased (OR = 0.542; 95% CI, 0.427-0.688; *P* < .001). Similar results were found for work-life balance (OR = 0.643; 95% CI, 0.559-0.739; *P* < .001), teamwork (OR = 0.525; 95% CI, 0.409-0.672; *P* < .001), and diversity (OR = 0.837; 95% CI, 0.710-0.985; *P* = .03).

The significance of variance of explained contributions indicated that EHR metrics accounted for 1.3% of model variance (*P* = .001) and work culture domains account for 17.6% of variance (*P* < .001). Interaction effect of sex to variables of interest was only significant for commitment and work-life, indicating that as the levels of these domains increased, the likelihood of burnout decreased more significantly for men compared with women (Figure 2).

## Discussion

The etiologies of clinician burnout are multifactorial and likely representative of a combination of the individual, local environment, regulatory requirements, and EHR technology.^22^ Our study describes the relationship of clinician burnout to EHR usage metrics and work culture across sex for attending physicians, APPs, and house staff.

We found that burnout was associated with commitment and work culture. Our multivariate analysis, taking into consideration clinician demographic characteristics, sex, EHR metrics, and wellness survey, found wellness domains suppressed the significance of EHR metrics for average patient age, total time in system, and in-basket messages. This suggests that wellness domains have greater explanatory power, which is consistent with the results of likelihoods test of *R^2^* difference and relative fit statistics.

The only EHR metric in our multivariate analysis to contribute significantly to burnout was number of days in the system. Interestingly, increasing days in the system were associated with a decreased likelihood of burnout (Table 3), potentially reflecting increased efficiency of usage of the EHR by clinicians for higher volume EHR users. Other EHR metrics derived as products of clinical care, such as length of notes or percentage of appointments closed the same day, did not differ significantly by sex.

Female clinicians reported more burnout than their male colleagues did across all 3 clinician types. These results support previous findings related to sex differences in burnout and EHR use metrics.^20,26^ While female clinicians spent more total time in the EHR and had more days with appointments, these measures did not lead to more clinician encounters or more total in-basket messages than male clinicians. The incongruence of the EHR time metric to volume metrics may be derivative of other workflow processes outside of the EHR to support clinicians that are not captured directly in the data. For example, some clinical workflows may allow other personnel to attach and complete in-basket metrics that would not be captured in the time spent completing messages.

Differences by sex in how clinicians deliver clinical care may also be driving these differences in EHR usage metrics.^22,42^ For example, female clinicians spend more time in direct patient care, even to the disadvantage of their overall volume of encounters.^18^ They may also be responding to different gendered expectations for care encounters reflected in the time spent in front of the patient.^24^ Regardless, Chen et al^43^ found trends of improved clinical quality of care also taking more time, thus validating the time spent.

All clinicians, regardless of sex or burnout category, spent approximately one-third of their total EHR usage after hours. After-hours time in the EHR has been of significant concern as a driver of burnout.^44^ The consistency of EHR use after hours and across all burnout categories appears to be more reflective of the flexibility to utilize the EHR at times that are more effective for them to complete their work. The relative decrease in after-hours time for female clinicians with moderate-to-severe burnout may be indicative of other competing priorities outside of work for these clinicians that necessitate improved efficiency with the EHR. Our results suggest that the time of day when a clinician works is not as important as the volume of time that they work.

Among the clinician groups, house staff shared the most similar work volume metrics for number of days in the system and days with appointments. These similarities can likely be attributed to larger Graduate Medical Education time constraints and training requirements. However, we still found differences in female house staff EHR time metrics, with increased total time and after-hours time. These findings were more consistent with female clinicians’ peers overall.

Our results found sex differences across clinician types for increased time spent and differences in clinical volume in the EHR for female clinicians. Our data set did not include a full-time–equivalency (FTE) metric, so normalization of work volume to overall encounter volumes cannot be determined. The differences for EHR usage metrics were most significant for attending physicians, less so for APPs, and generally not present for house staff, which is suggestive of potential variations in FTE.

### Limitations

There are a number of limitations to our study. Our sample included clinicians from only 1 academic institution. While the data are limited in originating from a single institution, this is counterbalanced by the size of our cohort and inclusion of multiple specialties in our multivariate analysis.

Attending physicians and APPs represented significant portions of active clinicians. A minority of the GME house staff (approximately 10%) participated in the organization survey and thus the house staff results are less generalizable. Overall, our results may not be as generalizable to other health systems owing to the contextual effect related to our EHR implementation and local work culture.

There are inherent limitations to using vendor EHR usage metadata. The Signal report consists of preprocessed summative data of the voluminous UAL Lite. As such, it represents a secondary data source of metadata of various activities in the EHR in varied formats for time (both by day and by activity), volume, and clinician panel demographic. Additionally, the data does not include delineation of metrics for clinicians who work concurrently in both outpatient and inpatient settings. We saw evidence of clinical crossover, with in-basket messages for some clinicians including hospital medical chart completion notifications.

For this study, we focused on metrics based upon total time and volume for consistency of comparisons across clinicians. Without a relative clinical FTE, understanding of volume EHR metrics is limited. We note that attending physicians especially can have significant variation in the timing of clinical duties with other responsibilities. Consequently, we only analyzed 1 month of EHR usage metrics vs averaged month-to-month data.

We developed secondary derivations of EHR metrics when the available measures were not specific for work volume or too granular for comparison across clinicians. For example, the SecondsPerCompletedMsg Denominator metric represents all completed messages for a month. Since these can be completed by other support staff, we calculated the total number of in-basket messages as more comparable of volume across clinicians. To limit introduction of errors and ensure the data used were representative of the metrics we calculated, definitions and data interpretation were cross-referenced with vendor representatives.

Finally, our study does not include patient outcomes. Without measures of potential value of EHR activities to the care of the patient, discrimination of the time and volume of work in the EHR cannot be fully assessed. Future research should include the combination of patient outcomes, measures of severity of illness in tandem with EHR usage metrics, sex, and measures of burnout.

## Conclusions

This study provides insight into variations of EHR usage by sex and across 3 types of clinicians. We found that clinician burnout was associated with commitment and local work culture factors. Burnout was greater for female clinicians irrespective of differences with male counterparts in EHR usage.

## Notes

Supplement. **eTable 1.** Selected EHR Usage Log Metrics, Definitions, and Signal Source Data **eTable 2.** Work Culture Survey Questions, Their Linked Domains, and Cronbach’s α Values

## References

[1] Rotenstein LS, Torre M, Ramos MA, . Prevalence of burnout among physicians: a systematic review. JAMA. 2018;320(11):1131-1150. doi:10.1001/jama.2018.12777 30326495 PMC6233645

[2] Dyrbye LN, Shanafelt TD, Sinsky CA, . Burnout among health care professionals: a call to explore and address this under recognized threat to safe, high-quality care. NAM Perspectives. Published July 5, 2017. Accessed March 15, 2021. https://nam.edu/burnout-among-health-care-professionals-a-call-to-explore-and-address-this-underrecognized-threat-to-safe-high-quality-care/

[3] Shanafelt TD, Hasan O, Dyrbye LN, . Changes in burnout and satisfaction with work-life balance in physicians and the general US working population between 2011 and 2014. Mayo Clin Proc. 2015;90(12):1600-1613. doi:10.1016/j.mayocp.2015.08.023 26653297

[4] Gardner RL, Cooper E, Haskell J, . Physician stress and burnout: the impact of health information technology. J Am Med Inform Assoc. 2019;26(2):106-114. doi:10.1093/jamia/ocy145 30517663 PMC7647171

[5] Tawfik DS, Scheid A, Profit J, . Evidence relating health care provider burnout and quality of care: a systematic review and meta-analysis. Ann Intern Med. 2019;171(8):555-567. doi:10.7326/M19-1152 31590181 PMC7138707

[6] Fahrenkopf AM, Sectish TC, Barger LK, . Rates of medication errors among depressed and burnt out residents: prospective cohort study. BMJ. 2008;336(7642):488-491. doi:10.1136/bmj.39469.763218.BE 18258931 PMC2258399

[7] Halbesleben JR, Rathert C. Linking physician burnout and patient outcomes: exploring the dyadic relationship between physicians and patients. Health Care Manage Rev. 2008;33(1):29-39. doi:10.1097/01.HMR.0000304493.87898.72 18091442

[8] West CP, Dyrbye LN, Erwin PJ, Shanafelt TD. Interventions to prevent and reduce physician burnout: a systematic review and meta-analysis. Lancet. 2016;388(10057):2272-2281. doi:10.1016/S0140-6736(16)31279-X 27692469

[9] Erickson SM, Rockwern B, Koltov M, McLean RM; Medical Practice and Quality Committee of the American College of Physicians. Putting patients first by reducing administrative tasks in health care: a position paper of the American College of Physicians. Ann Intern Med. 2017;166(9):659-661. doi:10.7326/M16-2697 28346948

[10] Dyrbye LN, Trockel M, Frank E, . Development of a research agenda to identify evidence-based strategies to improve physician wellness and reduce burnout. Ann Intern Med. 2017;166(10):743-744. doi:10.7326/M16-2956 28418518

[11] Han S, Shanafelt TD, Sinsky CA, . Estimating the attributable cost of physician burnout in the United States. Ann Intern Med. 2019;170(11):784-790. doi:10.7326/M18-1422 31132791

[12] American Medical Association. AMA calls for continued investment in effort to reduce burnout. American Medical Association website. Published February 22, 2019. Accessed March 15, 2021. https://www.ama-assn.org/press-center/press-releases/ama-calls-continued-investment-effort-reduce-burnout

[13] Kroth PJ, Morioka-Douglas N, Veres S, . Association of electronic health record design and use factors with clinician stress and burnout. JAMA Netw Open. 2019;2(8):e199609-e199609. doi:10.1001/jamanetworkopen.2019.9609 31418810 PMC6704736

[14] Downing NL, Bates DW, Longhurst CA. Physician burnout in the electronic health record era: are we ignoring the real cause? Ann Intern Med. 2018;169(1):50-51. doi:10.7326/M18-0139 29801050

[15] Shanafelt TD, Dyrbye LN, Sinsky C, . Relationship between clerical burden and characteristics of the electronic environment with physician burnout and professional satisfaction. Mayo Clin Proc. 2016;91(7):836-848. doi:10.1016/j.mayocp.2016.05.007 27313121

[16] Arndt BG, Beasley JW, Watkinson MD, . Tethered to the EHR: primary care physician workload assessment using EHR event log data and time-motion observations. Ann Fam Med. 2017;15(5):419-426. doi:10.1370/afm.2121 28893811 PMC5593724

[17] Sinsky C, Colligan L, Li L, . Allocation of physician time in ambulatory practice: a time and motion study in 4 specialties. Ann Intern Med. 2016;165(11):753-760. doi:10.7326/M16-0961 27595430

[18] Ganguli I, Sheridan B, Gray J, Chernew M, Rosenthal MB, Neprash H. Physician work hours and the gender pay gap—evidence from primary care. N Engl J Med. 2020;383(14):1349-1357. doi:10.1056/NEJMsa2013804 32997909 PMC10854207

[19] Tsugawa Y, Jena AB, Figueroa JF, Orav EJ, Blumenthal DM, Jha AK. Comparison of hospital mortality and readmission rates for Medicare patients treated by male vs female physicians. JAMA Intern Med. 2017;177(2):206-213. doi:10.1001/jamainternmed.2016.7875 27992617 PMC5558155

[20] Gupta K, Murray SG, Sarkar U, . Differences in ambulatory EHR use patterns for male vs. female physicians. NEJM Catalyst 2019;5(6). doi:10.1056/CAT.19.0690

[21] Tait SD, Oshima SM, Ren Y, . Electronic health record use by sex among physicians in an academic health care system. JAMA Intern Med. 2020;181(2):288-290.doi:10.1001/jamainternmed.2020.5036 33284311 PMC7851731

[22] Templeton K, Bernstein CA, Sukhera J, . Gender-based differences in burnout: issues faced by women physicians. NAM Perspectives. Published online May 30, 2019. doi:10.31478/201905a

[23] Linzer M, Harwood E. Gendered expectations: do they contribute to high burnout among female physicians? J Gen Intern Med. 2018;33(6):963-965. doi:10.1007/s11606-018-4330-0 29435727 PMC5975148

[24] Jamoom EW, Heisey-Grove D, Yang N, Scanlon P. Physician opinions about EHR use by EHR experience and by whether the practice had optimized its EHR use. J Health Med Inform. 2016;7(4):1000240.doi:10.4172/2157-7420.1000240 27800279 PMC5084912

[25] Shanafelt TD, Mungo M, Schmitgen J, . Longitudinal study evaluating the association between physician burnout and changes in professional work effort. Mayo Clin Proc. 2016;91(4):422-431. doi:10.1016/j.mayocp.2016.02.001 27046522

[26] Tran B, Lenhart A, Ross R, Dorr DA. Burnout and EHR use among academic primary care physicians with varied clinical workloads. AMIA Jt Summits Transl Sci Proc. 2019;2019:136-144. 31258965 PMC6568076

[27] Rule A, Chiang MF, Hribar MR. Using electronic health record audit logs to study clinical activity: a systematic review of aims, measures, and methods. J Am Med Inform Assoc. 2020;27(3):480-490. doi:10.1093/jamia/ocz196 31750912 PMC7025338

[28] Sexton JB, Frankel A, Leonard M, Adair KC. SCORE: assessment of work setting Safety, Communication, Operational Reliability, and Engagement. Technical report 19-5. Published May 14, 2019. Accessed March 16, 2021. https://www.hsq.dukehealth.org/files/2019/05/SCORE_Technical_Report_5.14.19.pdf

[29] Schwartz SP, Adair KC, Bae J, . Work-life balance behaviours cluster in work settings and relate to burnout and safety culture: a cross-sectional survey analysis. BMJ Qual Saf. 2019;28(2):142-150. doi:10.1136/bmjqs-2018-007933 30309912 PMC6365921

[30] Sexton JB, Adair KC, Leonard MW, . Providing feedback following Leadership WalkRounds is associated with better patient safety culture, higher employee engagement and lower burnout. BMJ Qual Saf. 2018;27(4):261-270. doi:10.1136/bmjqs-2016-006399 28993441 PMC5867443

[31] Adair KC, Quow K, Frankel A, . The improvement readiness scale of the SCORE survey: a metric to assess capacity for quality improvement in healthcare. BMC Health Serv Res. 2018;18(1):975. doi:10.1186/s12913-018-3743-0 30558593 PMC6296100

[32] Adair KC, Kennedy LA, Sexton JB. Three good tools: positively reflecting backwards and forwards is associated with robust improvements in well-being across three distinct interventions. J Positive Psychol. 2020;15(5):613-622. doi:10.1080/17439760.2020.1789707 PMC8294345 34295357

[33] Sexton JB, Adair KC. Forty-five good things: a prospective pilot study of the three good things well-being intervention in the USA for healthcare worker emotional exhaustion, depression, work-life balance and happiness. BMJ Open. 2019;9(3):e022695. doi:10.1136/bmjopen-2018-022695 30898795 PMC6475256

[34] Adair KC, Rodriguez-Homs LG, Masoud S, Mosca PJ, Sexton JB. Gratitude at work: a prospective cohort study of a web-based, single-exposure well-being intervention for healthcare workers. J Med Internet Res. 2020;22(5):e15562. doi:10.2196/15562 32406864 PMC7256751

[35] Wheeler DL, Vassar M, Worley JA, A reliability generalization meta-analysis of coefficient alpha for the Maslach Burnout Inventory. Educ Psychol Meas 2011;71(1):231–244. doi:10.1177/0013164410391579

[36] Schaufeli WB, Bakker AB, Hoogduin K, Schaap C, Kladler A. On the clinical validity of the Maslach Burnout Inventory and the burnout measure. Psychol Health. 2001;16(5):565-582. doi:10.1080/08870440108405527 22804499

[37] Hunter B, Fenwick J, Sidebotham M, Henley J. Midwives in the United Kingdom: levels of burnout, depression, anxiety and stress and associated predictors. Midwifery. 2019;79:102526. doi:10.1016/j.midw.2019.08.008 31473405

[38] Micek MA, Arndt B, Tuan WJ, . Physician burnout and timing of electronic health record use. ACI Open. 2020; 4(01):e1-e8. doi:10.1055/s-0039-3401815 PMC10553367 37800093

[39] Menard S. Applied Logistic Regression Analysis. 2nd ed. Sage Publications; 2002. doi:10.4135/9781412983433

[40] Menard, S. Coefficients of determination for multiple logistic regression analysis. *The American Statistician*. 2000;54(1):17-24.

[41] Akaike H. A new look at the statistical model identification. IEEE Transactions on Automatic Control. 1974;19(6):716-723. doi:10.1109/TAC.1974.1100705

[42] Wallis CJ, Ravi B, Coburn N, Nam RK, Detsky AS, Satkunasivam R. Comparison of postoperative outcomes among patients treated by male and female surgeons: a population based matched cohort study. BMJ. 2017;359:j4366. doi:10.1136/bmj.j4366 29018008 PMC6284261

[43] Chen LM, Farwell WR, Jha AK. Primary care visit duration and quality: does good care take longer? Arch Intern Med. 2009;169(20):1866-1872. doi:10.1001/archinternmed.2009.341 19901138

[44] Robertson SL, Robinson MD, Reid A. Electronic health record effects on work-life balance and burnout within the I3 population collaborative. J Grad Med Educ. 2017;9(4):479-484. doi:10.4300/JGME-D-16-00123.1 28824762 PMC5559244

