# Supplementary material for: Clinician Burnout Associated With Sex, Clinician Type, Work Culture, and Use of Electronic Health Records
Source: JAMA Netw Open. 2021 Apr 20;4(4):e215686. doi: 10.1001/jamanetworkopen.2021.5686 (PMC8058638; doi:10.1001/jamanetworkopen.2021.5686)
Supplement: Supplement. — eTable 1. Selected EHR Usage Log Metrics, Definitions, and Signal Source Data eTable 2. Work Culture Survey Questions, Their Linked Domains, and Cronbach’s α Values [file jamanetwopen-e215686-s001.pdf]

Supplemental Online Content

McPeek-Hinz E, Boazak M, Sexton JB, et al. Clinician burnout associated with sex, clinician type, work culture, and use of electronic health records. *JAMA Netw Open*. 2021;4(4):e215686. doi:10.1001/jamanetworkopen.2021.5686

eTable 1. Selected EHR Usage Log Metrics, Definitions, and Signal Source Data

eTable 2. Work Culture Survey Questions, Their Linked Domains, and Cronbach's α Values

This supplemental material has been provided by the authors to give readers additional information about their work.

© 2021 McPeek-Hinz E et al. *JAMA Network Open.*

On-line Only Supplement

1. Appendix A: Selected EHR Usage Log Metrics, Definitions, and Signal Source Data

2. Appendix B: Work Culture Survey Questions their Linked Domains and Cronbach's alpha values

© 2021 McPeek-Hinz E et al. *JAMA Network Open.*

Table 1e: Selected Access Log Metrics, Definitions, and Signal Source Data

	**Selected Metric**	**Definition**	**Signal Source of Data (number of metrics)**
Patient Metrics	Provider Average Patient Age	Average age of patients	AveragePatientAge_valApr (1)
EHR Use Metrics for Time	Days in the system	Number of days provider is in EHR during the month reporting period.	TimeinSystemperDay_denApr (1)
	Total Time in minutes in the EHR for month of April	Same	TimeinSystemperDay_numApr (1)
	Total time in EHR in minutes on Unscheduled Days	Total time in EHR on unscheduled days	TimeOnUnscheduledDays_numApr (1)
	Total time in System outside of Scheduled hours	Total time in EHR 30 minutes before first patient and 30 minutes after the last scheduled patient	TimeOutsideScheduledHours_numApr (1)
	Calculated Total Unscheduled Time	Sum of total time in system outside of scheduled hours –both same day and on nonscheduled Days. Consolidated metric	TimeOnUnscheduledDays_numApr + TimeOutsideScheduledHours_numApr (additional metric)
	Calculated Percentage of Unscheduled Time /Total Time in System	Ratio of unscheduled time in system to scheduled time in system.	"Unscheduled time" metric/ "Time in system" metric (additional metric)
In-basket Volume Metric	Calculated Total IB Messages received in the month	In basket messages received in a month. Included are a variety of messages from patient messages	Total of 109 metrics from Messages Received by category variable (109)

© 2021 McPeek-Hinz E et al. *JAMA Network Open.*

In-basket Volume Metric		to internal communication messages	
	Seconds per Message	Time in seconds to complete a message	SecondsperCompletedMessage_valApr (1)
Clinical Volume Metrics	Number of Days with appointments for month of April	Same	DayswithAppointments_numApr (1)
	Average Progress Note Length (number of characters)	Average progress note length.	ProgressNoteLength_valApr (1)
	Percentage of Notes Closed the Same day as the appointment	Same	PercentofAppointmentsClosedSameDay_valApr (1)
	Calculated Total Patient Visits for Month	Total patient visits (new, return, and consults) in the reporting period	Sum of "total new visits", "total consults", and sum of "established patient visits" (15)
	Calculated Total new visits Calculated	Total new visit encounters in the reporting period	Sum of new patient level of service numerators for 99201-99205 (5)
	Calculated Total consults Calculated	Total consult encounters in the reporting period	Sum of consult patient level of service numerators for 99241-99245 (5)
	Calculated Total established visits Calculated	Total established encounters in the reporting period	Sum of established level of service numerators for 99211-99215 (5)

© 2021 McPeek-Hinz E et al. *JAMA Network Open.*

Table 2e

	**Appendix B: Work Culture Survey Questions their Linked Domains and Cronbach's alpha values.***	
**Domain**	**Question**	**Cronbach's alpha**
Teamwork Climate	If I perceive a problem with quality, it is difficult to speak up in my department/work unit.	0.7
	Disagreements within my work unit/department are appropriately resolved (i.e., not who is right but what is best for the patient).	
	Dealing with difficult colleagues is consistently a challenging part of my job.	
	Communication breakdowns are common when my work unit/department interacts with other work unit/department	
Personal Burnout	Events in my work unit/department affect my life in an emotionally unhealthy way.	0.88
	I feel burned out from my work.	
	I feel fatigued when I get up in the morning and have to face another day on the job.	
	I feel frustrated by my job.	
	I feel I am working too hard on my job.	
Safety	In this work setting/department, it is easy to discuss errors.	0.53

© 2021 McPeek-Hinz E et al. *JAMA Network Open.*

	I would feel safe being treated here as a patient.	
Work-life Balance	(In the past week I) Worked through a day/shift without any breaks	0.62
	(In the past week I) Slept less than 5 hours in a night	
	(In the past week I) Changed personal/family plans because of work	
Belonging	I feel like I belong at Duke.	NA
Career Development	Duke provides career development opportunities.	0.71
	I know what is required of me to advance my career at Duke.	
Commitment	I would like to be working at Duke three years from now.	0.74
	I would recommend Duke as a great place to work to friends and family.	
Empowerment	I have a chance to use my strengths everyday at work.	0.66
	I am involved in decisions that affect my work.	
Management	My ideas and suggestions are seriously considered by the person I report to.	0.84
	I receive regular feedback from the person I report to on my performance.	
Diversity	The person I report to creates an environment of trust.	NA
	Employees from all backgrounds have equal opportunities to succeed in this work setting/department	NA

© 2021 McPeek-Hinz E et al. *JAMA Network Open.*

Wellbeing	I am aware of resources to support my well-being at Duke (e.g., Employee Health, Live for Life services, Patient Safety Center resilience courses, Personal Assistance Services, etc).	NA
Workplace Violence	Verbal threats, harassment, or instances of physical violence by patients and visitors occur in my work-setting	NA
	Duke effectively manages instances of verbal threats, harassment, or instances of physical violence by patients and visitors that occur in my work-setting (CONTINGENT QUESTION: Only provide answer if response to Q27 is Rarely/Occasionally/Freq/Very Freq)	NA
Leadership Access and Responsiveness	The actions of the person that I report to demonstrate this organization's mission and values.	0.95
	The person I report to acts on new ideas.	
	The person I report to creates an environment of trust.	
	The person I report to has regular conversations with me about my performance.	
	The person I report to provides recognition for employees who do a good job.	
	The person I report to addresses issues in a timely manner.	
	The person I report to cares about quality improvement.	
	The person I report to consistently reinforces safe work practices.	
	The person I report to rounds with employees on a regular basis.	
	The person I report to is an effective communicator.	

© 2021 McPeek-Hinz E et al. *JAMA Network Open.*

	Senior leaders round with employees on a regular basis.	
	I feel comfortable discussing important workplace issues with the person to whom I report.	
Autonomy	Nurses have the authority to make clinical patient care decisions.	0.89
	Nurses are involved in decision-making outside of their immediate work unit.	
	Nurses are able to provide input into decision-making regarding organization or system polices and procedures.	
	Nurses have the opportunity to provide input into decisions regarding their broader work environment.	
	Nurses have the opportunity to provide input into decisions that impact patient care processes and procedures.	
Interprofessional Relationships	This organization values associates from different backgrounds.	0.86
	This organization treats associates with respect.	
	My supervisor creates an environment of trust.	
	Members of my work group treat one another with respect.	
	I enjoy working with my coworkers.	
	I can openly express my concerns to management at this organization.	
	My department works well together.	
	The physicians and nurses here work together as a well-coordinated team.	

© 2021 McPeek-Hinz E et al. *JAMA Network Open.*

Professional Development	I have the resources I need to grow and develop my career.	0.87
	I receive useful coaching and feedback from my supervisor.	
	In the last year, I have had opportunities at work to learn and grow.	
	I get the training I need to do my job effectively.	
	I receive appropriate feedback about my performance.	
Adequacy of Resources and Staffing	I have the tools and resources I need to work effectively.	0.75
	There is adequate staffing in my work area to provide excellent service.	
	I have sufficient time to provide the best care for our patients.	
	The physical conditions (light, heat, space, appearance) in my area are well-maintained.	
	*Note: Questions were set to Likert responses of "strongly disagree to strongly agree)	

© 2021 McPeek-Hinz E et al. *JAMA Network Open.*
